# Prediction of Long-Term Restenosis After Carotid Endarterectomy Using Quantitative Magnetic Resonance Angiography

**DOI:** 10.3389/fneur.2022.862809

**Published:** 2022-06-30

**Authors:** Lukas Andereggen, Sepideh Amin-Hanjani, Jürgen Beck, Markus M. Luedi, Jan Gralla, Gerrit A. Schubert, Angelo Tortora, Robert H. Andres, Marcel Arnold, Andreas Raabe, Michael Reinert

**Affiliations:** ^1^Department of Neurosurgery, Kantonsspital Aarau, Aarau, Switzerland; ^2^Faculty of Medicine, University of Bern, Bern, Switzerland; ^3^Department of Neurosurgery, The University of Illinois at Chicago, Chicago, IL, United States; ^4^Departments of Neurosurgery, Inselspital, Bern University Hospital, University of Bern, Bern, Switzerland; ^5^Department of Neurosurgery, Medical Center, University of Freiburg, Freiburg, Germany; ^6^Department of Anaesthesiology, Inselspital, Bern University Hospital, University of Bern, Bern, Switzerland; ^7^Department of Neuroradiology, Inselspital, Bern University Hospital, University of Bern, Bern, Switzerland; ^8^Department of Neurology, Inselspital, Bern University Hospital, University of Bern, Bern, Switzerland; ^9^Clinic for Neurosurgery, Hirslanden Klinik St. Anna, Lucerne, Switzerland

**Keywords:** cerebral blood flow, carotid endarterectomy, quantitative phase-contrast MR angiography, restenosis, carotid artery stenosis

## Abstract

**Background:**

To detect restenosis after carotid endarterectomy (CEA), long-term monitoring is required. However, non-selective follow-up is controversial and can be limited by costs and logistical considerations.

**Objective:**

To examine the value of immediate perioperative vessel flow measurements after CEA using quantitative magnetic resonance angiography (QMRA) to detect patients at risk of long-term restenosis.

**Methods:**

A prospective cohort study with long-term sonographic follow-up after CEA for symptomatic internal carotid artery stenosis (ICAs) > 50%. In all patients, vessel flow has been assessed both pre- and postoperatively using QMRA within ±3 days of surgery. Data on QMRA assessment were analyzed to identify patients at risk of restenosis for up to 10 years.

**Results:**

Restenosis was recorded in 4 of 24 patients (17%) at a median follow-up of 6.8 ± 2.6 years. None of them experienced an ischemic event. Perioperative flow differences were significantly greater in patients without long-term restenosis, both for the ipsilateral ICA (*p* < 0.001) and MCA (*p* = 0.03), compared to those with restenosis (*p* = 0.22 and *p* = 0.3, respectively). The ICA mean flow ratio (*p* = 0.05) tended to be more effective than the MCA ratio in predicting restenosis over the long term (*p* = 0.35).

**Conclusion:**

Our preliminary findings suggest that QMRA-based mean flow increases after CEA may be predictive of restenosis over the long term. Perioperative QMRA assessment could become an operator-independent screening tool to identify a subgroup of patients at risk for restenosis, in whom long-term monitoring is advised.

## Introduction

Restenosis in patients who undergo surgery for symptomatic internal carotid artery stenosis (ICAs) has been reported in up to 36% of patients during long-term follow-up (1, 2). While periodic postoperative sonography remains the gold standard in the detection of recurrent stenosis (3), indiscriminate screening has proved to be costly and inefficient (4). Given that a subset of patients may benefit from frequent and continuous monitoring after revascularization (5), early identification of patients at risk of restenosis over the long term is warranted.

Different imaging techniques have been used to assess for restenosis (6), with quantitative magnetic resonance angiography (QMRA) enabling quantification of the blood flow rate (ml/min) in multiple extra- and intracranial arteries simultaneously, making it a promising technique for hemodynamic investigation in patients with carotid artery diseases (7–10). In this study, we aimed at evaluating whether perioperative flow measurements using QMRA can identify subgroups of patients at risk of restenosis, in whom closer long-term monitoring might be warranted.

## Methods

### Study Design

We assessed patients undergoing QMRA before and after carotid endarterectomy (CEA) for ICAs > 50%, in whom long-term (≥24 months) sonography follow-up was recorded. The records were prospectively maintained from January 2011 to December 2020. Patients presenting with a new ischemic stroke or a transient ischemic attack and corresponding carotid stenosis ≥ 50% were admitted to our tertiary stroke referral center (further details of the criteria for enrollment are provided in the Supplementary Materials). A preoperative neurological examination, routine blood analysis, and a 12-lead electrocardiogram (ECG) were performed. The degree of carotid stenosis was measured by sonography according to the North American Symptomatic Carotid Endarterectomy Trial (NASCET) criteria (11).

### Sonographic Studies

Sonographic results at the baseline and last follow-up visits were recorded. The surveillance protocol consisted of a 6-month postoperative sonographic study and–if unsuspicious–an annual routine follow-up examination to detect restenosis. Arteries were examined with a linear-array transducer (9 MHz) for extracranial examination and a low-frequency phased-array transducer (2 MHz) for transtemporal insonation in the axial plane (8). Restenosis at the site of CEA was defined as recurrent luminal narrowing, with the percentage of stenosis being calculated according to the NASCET criteria (11).

### QMRA Studies

A 3T MRI (MagnetomVerio, Siemens, Erlangen, Germany) equipped with a 12-channel head coil and a 4-channel neck coil was used (12, 13). The 3D time-of-flight MR angiography of the arteries was assessed, and rotating 3D surface-rendered vascular images were reconstructed. Quantification of blood flow using the gated fast 2D phase-contrast sequence was calculated from Non-Invasive Optimal Vessel Analysis (NOVA) software (VasSOL, Chicago, IL, USA) (14). This protocol has been used routinely for the evaluation of patients with cerebrovascular diseases (7, 8, 14). About 30 min, in addition to the MRI scan time, was used for the NOVA examination, depending on the complexity of the vessels studied. The end-tidal partial pressure of carbon dioxide (EtCO_2_) and patients' blood pressure was registered during the QMRA studies. Time points of QMRA measurements before and after CEA were noted. Flow measurements assessed by QMRA in ml/min were not revealed until the completion of data entry into the database by an independent investigator.

### Surgical Procedure

A microscope-assisted non-patch endarterectomy technique not affecting imaging quality was used (15). All the patients were preoperatively treated with anti-platelet agents. CEA was performed under general anesthesia. Neuromonitoring was performed throughout the procedure using an intraoperative EEG monitor, transcranial Doppler (TCD), and somatosensory-evoked potentials (SSEP). Before cross-clamping, a burst-suppression EEG pattern was implemented with propofol. Intravenous heparin (100 U/kg) was administered prior to ICA exposure. After cross-clamping, the stenotic segment of the ICA was incised, the endarterectomy was completed, and the arteriotomy was sealed, with a 6–0 monofilament continuous suture. If the mean TCD flow velocity was <50% of the pre-clamping values without restoration through blood pressure increase, or in the case of SSEP deterioration, an intraluminal shunt was used. After clamp release, blood flow was controlled with a micro doppler probe. Three hundred mg of acetylsalicylic acid was administered intravenously 6 h after surgery. As for follow-up therapy, antiplatelet drugs and treatment of vascular risk factors with optimal medical therapy were standard of care (16).

### Statistical Analyses

Data were analyzed using IBM SPSS statistical software Version 24.0 (IBM Corp., New York, NY, USA) and GraphPad Prism (V7.04 software, San Diego, CA, USA). The Shapiro–Wilk test was applied to verify normal distribution. Continuous variables are expressed as mean ± SD (normally distributed data), or as median values and interquartile range (IQR, 25th to 75th percentile), respectively. Categorical variables are given as numbers and percentages. Differences between the normally distributed data of 2 groups were analyzed using the paired *t*-test, and the Wilcoxon signed-rank test was used to analyze non-parametric data. Due to the number of patients studied, predictors for restenosis were assessed using contingency tables. A significance level of *p* ≤ 0.05 was applied.

## Results

### Patient Demographics

QMRA was introduced from November 2011 to December 2013 in our institution, with 153 patients having CEA for ICA stenosis during this period. Twenty-five of those patients were randomly selected for pre- and postoperative QMRA examination. Long-term sonographic follow-up data were finally available for 24 patients with symptomatic ICAs, with one patient lost during follow-up. Baseline characteristics are summarized in Table 1. There were no statistically significant differences between the patients with and without restenosis about baseline characteristics. The cohort consisted of 6 women and 18 men, with a mean age (±SD) of 69 ± 9 years. Thirteen patients were ≥70 years old. Cardiovascular risk factors were noted as follows: hypertension (79%), diabetes mellitus (29%), dyslipidemia (79%), active smoking (67%), peripheral artery occlusive disease (17%), positive family history of cerebrovascular diseases (21%), and obesity (i.e., BMI ≥ 30 kg/m^2^) in 4 (17%) patients. High-grade stenosis ≥ 90% was present in 8 patients (33%). QMRA was performed within 1.6 ± 1.2 days of surgery, specifically 1.2 ± 1.5 days before and 1.9 ± 0.9 days after surgery.

**Table 1:** Clinical predictors of restenosis over the long-term.

**Clinical characteristics, *n* (%)**	**No stenosis**	**Restenosis**	**All patients**	***P*-value**
Total cases	20 (83)	4 (17)	24 (100)	
Age, years (mean ± SD)	70.5 ± 9.3	66.3 ± 6.6	69.8 ± 8.9	0.4
Sex (female)	6 (30)	0 (0)	6 (25)	0.54
Hypertension according to AHA	16 (80)	3 (75)	19 (79)	1
Diabetes mellitus	7 (35)	0 (0)	7 (29)	0.28
Dyslipidemia	16 (80)	3 (75)	19 (79)	1
Active smoking	13 (65)	3 (75)	16 (67)	1
Obesity (BMI > 30 kg/m^2^)	3 (15)	1 (25)	4 (17)	0.54
BMI (mean ± SD)	26.4 ± 3.7	24.9 ± 3.8	26.2 ± 3.6	0.57
Coronary artery disease	7 (35)	1 (25)	8 (33)	1
Peripheral vascular disease	3 (15)	1 (25)	4 (17)	0.54
Positive family history of cerebrovascular disease	4 (20)	1 (25)	5 (21)	1
Presenting symptom—TIA	12 (60)	4 (100)	16 (68)	0.26
Presenting symptom—stroke	8 (40)	0 (0)	8 (33)	0.26
Follow-up, months (mean ± SD)	82.5 ± 32.4	81.0 ± 30.7	81.0 ± 30.7	0.89

*Unless otherwise noted, parameter values are given as n (%). AHA, American Heart Association; BMI, body mass index; BP, blood pressure; ICA, internal carotid artery; MCA, middle cerebral artery; SD, standard deviation; TIA, transient ischemic attack*.

At a mean (±SD) follow-up of 81 ± 31 months (range, 24–110 months), restenosis was noted in 4 patients (17%). Thereby, mild (50%) restenosis was present in 2 patients, moderate (50–69%) stenosis in 1 patient, and high-grade restenosis (≥70%) in 1 patient. Carotid stenting was subsequently performed in the patient with high-grade stenosis. None of the patients with restenosis experienced a new neurological deficit in the follow-up period.

**Figure 1:**
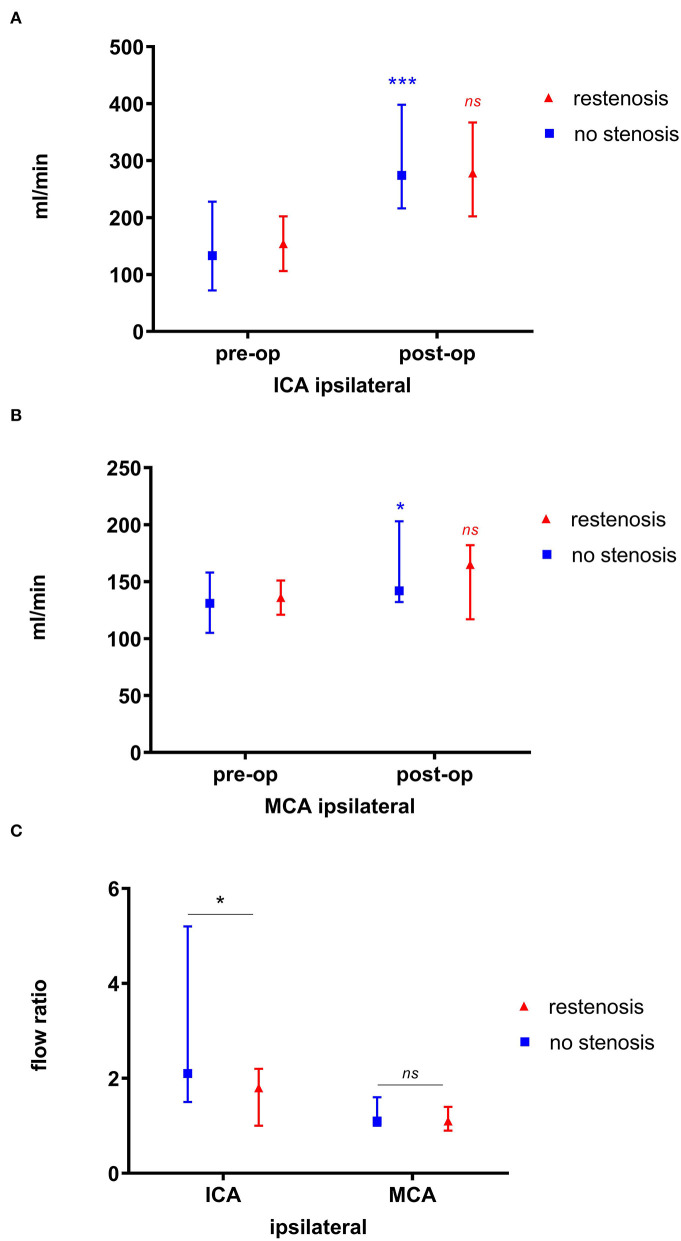
Perioperative blood flow values in patients with non-patch CEA. Significant perioperative flow differences are observed in patients without restenosis vs. those with restenosis over the long term, both in the ipsilateral ICA **(A)** and the MCA **(B)**. The ICA median flow ratio (*p* = 0.05) tended to be more efficient than the MCA ratio in predicting restenosis (*p* = 0.35) **(C)**. *significant; ***highly significant (*p* < 0.001); ns, non-significant.

QMRA results in patients with and without restenosis are summarized in Tables 2, 3. In patients without long-term restenosis, perioperative flow differences (postoperative flow – preoperative flow) were significantly higher than in patients with restenosis, both in the operated ICA (274 ml/min; IQR 216–398 ml/min) vs. 133 ml/min (IQR 72-228 ml/min), *p* < 0.001 (Figure 1A), and ipsilateral MCA (142 ml/min (IQR 132–203 ml/min) vs. 131 ml/min (IQR 105–158), *p* = 0.03 (Figure 1B), respectively. In contrast, no significant differences in the perioperative flow were observed in the patients with restenosis over the long term (*p* = 0.22 for ICA, and *p* = 0.3 for MCA; Figures 1A,B, respectively). The perioperative median flow ratio (postoperative flow value/preoperative flow value) was significantly higher in the patients without restenosis vs. those with restenosis over the long term. This was true in the operated ICA [2.1 (IQR, 1.5–5.2) vs. 1.8 (IQR, 1.0–2.2); *p* = 0.05], but not the ipsilateral MCA [1.1 (IQR 1.0–1.6) vs. 1.1 (IQR, 0.9–1.4), *p* = 0.35; Figure 1C]. Hence, the ICA ratio (*p* = 0.05) tended to be more effective than the MCA ratio in predicting long-term restenosis (*p* = 0.35). Perioperative flow differences in the ipsilateral ICA were noted to be 157 ml/min (IQR, 76–214 ml/min). Thereby, perioperative flow differences were not significantly different in the patients presenting with ≥90% ICA stenosis compared to those with <90% ICA stenosis; 134 ml/min (IQR, 29–182 ml/min) vs. 167 ml/min (IQR, 92–241 ml/min, *p* = 0.23). Likewise, the perioperative median flow ratio was not different in the patients with ≥90% ICA stenosis vs. those with <90% ICA stenosis; ICA, 2.1 (IQR, 1.2–3.3) vs. 1.9 (IQR, 1.4–5.8; *p* = 0.54). Comparing the patients with ≥70% stenosis, we noted the following results: perioperative flow differences were not significantly different in the patients presenting with ≥70% ICA stenosis compared to those with <70% ICA stenosis; 154 ml/min (IQR, 12–182 ml/min) vs. 173 ml/min (IQR, 102–233 ml/min, *p* = 0.16). Likewise, the perioperative median flow ratio was not different in patients with ≥70% ICA stenosis vs. those with <70% ICA stenosis; ICA 2.0 (IQR 1.1–3.1) vs. ICA 2.1 (IQR 1.5–7.5).

**Table 2:** Radiological predictors for restenosis over the long-term.

**Radiological characteristics**	**Side**	**Vessel**	**No stenosis**	**Restenosis**	**All patients**	***P*-value**
Stenosis grading in % (mean ± SD)			83.8 ± 4.6	85 ± 10.8	84 ± 5.7	0.83
Plaque length in mm (mean ± SD)			21.1 ± 2	19.8 ± 6.7	20.8 ± 3.1	0.73
Flow preoperative	Ipsilateral	ICA	133 (72–228)	154 (10–6202)	142 (75–215)	0.99
Flow postoperative			274 (216–398)	278 (202–367)	274 (215–387)	0.43
Flow differences			160 (81–219)	114 (4–224)	157 (76–214)	0.26
Median flow ratio			2.1 (1.5–5.2)	1.8 (1.0–2.2)	2.0 (1.4–3.8)	0.05
Flow preoperative	Ipsilateral	MCA	131 (105–158)	136 (121–151)	131 (110–155)	0.69
Flow postoperative			142 (132–203)	165 (117–182)	152 (132–184)	0.49
Flow differences			22 (12–55)	19 (9–50)	22 (12–55)	0.25
Median flow ratio			1.1 (1.0–1.6)	1.1 (0.9–1.4)	1.1 (1.0–1.5)	0.35

*Unless otherwise noted, parameter values are given as median (IQR)*.

*ICA, internal carotid artery; MCA, middle cerebral artery; SD, standard deviation*.

**Table 3:** QMRA-assessed blood flow values before and after CEA.

**Restenosis**	**Side**	**Vessel**	**Preoperative (median, IQR)**	**Postoperative (median, IQR)**	**Difference (median, IQR)**	***P*-value**
No	Ipsilateral	ICA	133 (72–228)	274 (216–398)	160 (81–219)	<0.001
Yes			154 (106–202)	278 (202–367)	114 (4–224)	0.22
No		MCA	131 (105–158)	142 (132–203)	22 (12–55)	0.03
Yes			136 (121–151)	165 (117–182)	19 (9–50)	0.3
No	Contralateral	ICA	283 (239–317)	228 (197–280)	72 (30–109)	0.12
Yes			233 (160–306)	209 (182–236)	24 (22–26)	0.63
No		MCA	131 (105–158)	142 (132–203)	18 (11–40)	0.14
Yes			136 (121–151)	165 (117–183)	35 (15–47)	0.13

*CEA, carotid endarectomy; ICA, internal carotid artery; MCA, middle cerebral artery; IQR, interquartile range*.

In Spearman's rank correlation, we found a positive association between perioperative flow differences and the following risk factors for restenosis associated in CREST (5): smoking (*r* = 0.47, *p* = 0.03), diabetes (*r* = 0.22, *p* = 0.33), dyslipidemia (*r* = 0.1, *p* = 0.71), and female sex (*r* = 0.416, *p* = 0.05). Likewise, we found a positive association between the median flow ratio and smoking (*r* = 0.485, *p* = 0.02), diabetes (*r* = 0.223, *p* = 0.318), dyslipidemia (*r* = 0.01, *p* = 0.96), and female sex (*r* = 0.4, *p* = 0.06).

### Mortality and Morbidity

None of the patients died. Postoperatively, two patients had a tonic-clonic seizure, and one patient had a refractory headache, potentially related to cerebral hyperperfusion. No postoperative hemorrhage or wound infections were noted.

## Discussion

Our preliminary findings indicate that QMRA-based mean flow increases after CEA may be predictive of restenosis over the long term, with significantly higher perioperative flow rate increases in the operated ICA being noted in patients without restenosis vs. those with restenosis. The underlying mechanism for the inverse relationship between the higher flow differences and long-term restenosis needs to be further elucidated. As the degree of stenosis has often been shown to be inversely related to the length of a stenosis (17), we hypothesize that following CEA in those patients with longer stenosis segments might stimulate neointimal hyperplasia and thus restenosis formation more frequently. Although we did not investigate the significance of the length of stenosis in this study, the relation of the excess restenosis rate increases with longer stenosis at the baseline (18). Patients with long stenoses may be at increased risk of restenosis, given the longer endothelial and plaque surface prone to atherosclerotic debris reformation. Furthermore, the incidence of residual stenosis enhances restenosis rates (19). As smaller perioperative flow changes have been present in those with restenosis formation, we might hypothesize that following CEA residual stenosis might have been greater in them. Namely, the plaque length and thickness were significantly higher in the residual stenosis group than in the non-residual stenosis group in a recent analysis (18). In addition, as our results show a significant positive association between female sex and perioperative flow differences, female sex was related to an increased risk of restenosis in the CREST trial (20), suggesting that the width of intimal hyperplasia in women with smaller-diameter carotid arteries might lead to a greater degree of restenosis.

A restenosis rate of 17% was observed after a median follow-up period of ≈7 years, corroborating previous findings that reported restenosis rates to range from 3 to 36% (5, 15). The relatively higher incidence of restenosis might reflect i) the fact that the majority of the patients in our cohort showed a mild-to-moderate reduction of the carotid diameter, and ii) the long-term follow-up of this study. Restenosis ≥ 70% requiring stenting was detected in one patient (4%), which is in line with previous studies reporting restenosis rates of around 3% during long-term follow-up (21).

Multiple studies have identified clinical and morphological variables that potentially predict restenosis after carotid surgery. Patients with delayed CEA after stroke have been associated with increased risk (2), while patch closure has been shown to reduce restenosis after CEA (22). In addition, carotid plaque features have been associated with increased restenosis after CEA (23, 24). In a secondary analysis of the CREST trial, female sex, diabetes, and dyslipidemia were independent predictors of restenosis after both carotid artery stenting and CEA, while smoking was associated with an increased likelihood of restenosis after CEA (5). Smoking and female sex were associated with QMRA-assessed perioperative blood flow changes. As increases in QMRA-based mean flow after CEA were predictive of restenosis over the long term, there may be an increased likelihood of restenosis, particularly in female patients and active smokers, harboring smaller perioperative flow differences. Interactions between these risk factors and flow changes should be examined in future research to provide a more comprehensive context to this relationship.

Given that non-selective long-term monitoring is known to be costly and inefficient, in particular when the postoperative sonographic result is normal (25), as in our cohort, a subset of patients may still benefit from frequent and continuous monitoring after revascularization (5). Hence, early identification of patients at risk of restenosis over the long term would be useful.

QMRA is an established, operator-independent, non-invasive tool that makes hemodynamic investigations possible in patients with carotid artery stenosis (7, 8, 14). Previous studies have revealed the feasibility of using QMRA in the detection of intracranial in-stent stenosis, or the identification of stenosis in extra-intracranial bypass surgery, Moyamoya angiopathy, or intracranial hypertension (9, 14, 26–29). However, as far as we know, our study is the first in which QMRA-assessed values of perioperative flow were examined about subsequent detection of long-term restenosis. Identification of patients with a high risk of long-term restenosis, therefore, would identify a subgroup early on in whom continuous monitoring is indicated. As our preliminary data show, QMRA may have the potential to identify such a subgroup of patients, in particular those with lower perioperative mean flow increase ratios.

### Study Limitations

Our study is limited by the small sample size, which precludes the assessment of independent predictors for restenosis using multiple logistic regression. Related costs of QMRA examination may limit its use over a prolonged period in the perioperative period. On the other hand, the potential benefits of being able to select patients at risk over the long term might justify its application on a routine basis. Potential drawbacks of QMRA assessments are the technique's sensitivity to patient movement, particularly in the postoperative period in older, less compliant patients. In addition, tortuous vessels (i.e., middle cerebral arteries) could have affected the measured value of flow when using 2D phase-contrast MRA. Prospective validation of our findings in a larger cohort would be an important step in confirming the utility of QMRA for the detection of patients at risk of restenosis over the long term.

## Conclusion

Our preliminary findings suggest that QMRA-based mean flow increases occurring after CEA may be predictive of restenosis over the long term. Perioperative QMRA assessment could potentially become an operator-independent screening tool for identifying patients at risk of restenosis, for whom regular monitoring is advised.

## Data Availability Statement

The original contributions presented in the study are included in the article/Supplementary Material, further inquiries can be directed to the corresponding author.

## Ethics Statement

The Human Research Ethics Committee of Bern (Kantonale Ethikkommision KEK Bern, Bern, Switzerland) approved the study (KEK 30/02). The study was performed in accordance with the ethical standards laid down in the 1964 Declaration of Helsinki and its later amendments. The ethics committee waived the need for informed consent for this study as part of the study approval.

## Author Contributions

LA contributed to the study concept and design, data collection, statistical analysis and interpretation, drafting of the manuscript, critical revision, and final approval of the article. SA-H and MR contributed to data analysis and interpretation, critical revision, and final approval of the article. JB, JG, GS, AT, RA, MA, ML, and AR contributed to critical revision and final approval of the article. All authors contributed to the article and approved the submitted version.

## Conflict of Interest

The authors declare that the research was conducted in the absence of any commercial or financial relationships that could be construed as a potential conflict of interest.

## Publisher's Note

All claims expressed in this article are solely those of the authors and do not necessarily represent those of their affiliated organizations, or those of the publisher, the editors and the reviewers. Any product that may be evaluated in this article, or claim that may be made by its manufacturer, is not guaranteed or endorsed by the publisher.

## Abbreviations

CEA: carotid endarterectomy
ICA: internal carotid artery
MCA: middle cerebral artery
MR: magnetic resonance
QMRA: quantitative phase contrast MR angiography.
