# Supplementary material for: Prediction of Long-Term Restenosis After Carotid Endarterectomy Using Quantitative Magnetic Resonance Angiography
Source: Front Neurol. 2022 Jun 30;13:862809. doi: 10.3389/fneur.2022.862809 (PMC9279565; doi:10.3389/fneur.2022.862809)
Supplement: Supplementary file 1 [file Table_1.DOCX]

**SUPPLEMENTAL MATERIAL**

**Prediction of long-term restenosis after carotid endarterectomy using quantitative magnetic resonance angiography**

Lukas Andereggen^1,2^; Sepideh Amin-Hanjani^3^; Jürgen Beck^2,8^; Jan Gralla^5^; Gerrit A. Schubert^1^; Angelo Tortora^1^; Robert H. Andres^2,4^; Marcel Arnold^6^; Markus M. Luedi^7^; Andreas Raabe^4^; Michael Reinert ^2,9^

^1^Department of Neurosurgery, Kantonsspital Aarau, Aarau, Switzerland

^2^Faculty of Medicine, University of Bern, Bern, Switzerland

^3^Department of Neurosurgery, University of Illinois at Chicago, Chicago, Illinois, USA

^4^Department of Neurosurgery, ^5^Neuroradiology, ^6^Neurology, and ^7^Anaesthesiology, Inselspital, Bern University Hospital, University of Bern, Bern, Switzerland

^8^Department of Neurosurgery, Medical Center, University of Freiburg, Freiburg, Germany

^9^Neurocenter Central Switzerland, Hirslanden Klinik St. Anna, Luzern, Switzerland

**Correspondence:**

Lukas Andereggen, MD

Department of Neurosurgery

Kantonsspital Aarau,

5000 Aarau, Switzerland

E- Mail: lukas.andereggen@gmail.com

Phone +41 62 838 66 90

Fax: +41 62 838 66 29

Orcid ID: 0000-0003-1764-688X

**Supplemental Methods**

***Eligibility criteria****

**Inclusion criteria**

Inclusion criteria for QMRA examination were age ≥ 18 years and modified Rankin Scale score <3. Study inclusion criteria were QMRA examination before and after surgery within ≤4 days and long-term follow-up sonography for assessment of restenosis.

**Exclusion criteria**

Patients with atrial fibrillation, severe aphasia or altered mental status confounding the QMRA examination, pregnancy, or medical ferromagnetic devices precluding MRI examination were excluded. In addition, patients with non-atherosclerotic ICAs, including vessel dissection, fibromuscular dysplasia, vasculitis, or radiation-induced vasculopathy, were not enrolled.
